# Rubeola

**Published:** 1861-07

**Authors:** 


### MERCY HOSPITAL—MEDICAL WARDS.

Service Prof. N. S. DAVIS, M. D., Visiting Physician.

*Reported for the* EXAMINER.

I. *Rubeola.*—Owing to the want of accommodations at their barracks, some fifteen or twenty members of the Irish Brigade have been admitted into the Hospital during the month, suffering from the measles, and in some cases complicated with other diseases,—gonorrhæa, pueumonia, diarrhæa, etc. Ample opportunity has thus been furnished students to become familiar with the diagnosis and treatment of this usually simple malady. *Usually* simple—but owing to its liability to be incorrectly diagnosed, as in more than one case under the lecturer's own observation, where it has been mistaken for *variola*,—or from its tendency to occur as an epidemic, as in the present instance, and as in nearly all our camps, worthy more than a mere passing mention.

The first stages of the *Eruptive Fevers* are not unlike those of any idiopathic fever, except in their duration. There is the same uncomfortable feeling, with a disposition to stretch and yawn, and an indisposition to exertion; a want of elasticity and animation, the same fugitive aches, in the head, in the limbs, in the back or body, the bad taste in the mouth, the general languor and enervated condition of the muscular system, alike preceding the periodical, the typhoid, and the eruptive fevers. In the first, these symptoms may last only two or three days before the occurrence of the premonitory chill, followed by a well-marked febrile movement and the characteristic remissions or intermissions. In the typhoid class, the chill will usually be omitted, but the period between the first uncomfortable feelings and the actual yielding to the attack and taking to bed, will be lengthened to a week or more—and even then your patient may not feel very sick on his bed, but may fancy himself able to be about his ordinary occupations, until he tries to make some continued exertion.

The onset of the Eruptive Fevers, on the contrary, is, if pure and uncomplicated, abrupt and well-marked; commencing with a distinct febrile movement, preceded by a half-hour or so of chilliness—not a marked chill—during which the patient finds the fire agreeable, but soon feels flushes of heat alternating with his slight chills, which flushes soon develop into a fever, accompanied by thirst, heat of skin, accelerated pulse, headache, bad tasting mouth, loss of appetite, etc. This difference in the duration of the premonitory symptoms, furnishes the first guide to a differential diagnosis, as between the three classes mentioned.

Recollect that the patient is attacked with fever aside from the local eruption, which fever is to be distinguished from periodical fevers by absence of any marked chill, from continued fevers by the rapidity of its progress; in a word, that it has the suddenness of the periodical fever without its chill.

To diagnose between the varieties of exanthems, however, is, in the early stage, a more difficult and delicate task, and more especially so before the eruption has made its appearance—indeed, when the eruption is marked and full the diagnosis to the intelligent student, is comparatively easy. The characteristic of Measles is its *cough*—hard, dry, harsh and hollow, incessant and sometimes so violent as to considerably exhaust the patient; with this there is soreness and pain in the throat and thorax behind the sternum; there is huskiness of voice, even to complete aphonia, as in *No. ten;* there is sneezing, and sometimes almost uncontrollable epistaxis, as in *No. five*, who has lost not less than thirty-six to forty ounces of blood during the last forty-eight hours; there is every symptom, in short, of a congested, irritable condition of the mucous surfaces of the head and chest generally; the eyes are red and watery, and the cellular tissue below the eye swollen and infiltrated, while exposure to the light is painful. The patient will tell you he has taken a violent cold, and all his symptoms are such as to induce him to such a belief. But about the fourth day after the commencement of the attack, the appearance] of a *rash*, as it is termed, determines the question. Up to the second day, some doubts may be entertained as to whether the disease be not scarlatina; but if we remember that the rash or eruption in this appears pretty nearly uniformly on the second day,—that there is no cough or coryza, that the pulse is very rapid, this being a marked feature,—the pulse frequently attaining 120, and out of proportion to the amount of fever,—if we further remember that scarlatina is marked by the swelling and a bright scarlet flush of the fauces, palate, uvula and tonsils, by tumefaction of the lymphatic glands of the neck and jaws, and not unfrequently by nausea,—if we keep these facts in mind, it will be easy to avoid the alarm and anxiety which the announcement of this household scourge never fails to carry to the parent.

If the fever, however, continues another day, or more than forty-eight hours from its commencement, without eruption, if there be severe headache, much nausea, intolerable pain in the lumbar region, a peculiarly flushed face, much depression of spirits, or delirium, or stupor, with the pulse about 100, and the rash appearing within the first seventy-two hours after the febrile symptoms, small-pox may be pretty confidently looked for.

Called to a patient in the eruptive stage, and it is then that you will most likely be sent for, you will learn something from the attendants on the points already mentioned; but a careful examination of the eruption itself will now form your most trustworthy guide. In scarlatina the rash or effloresence will be found of a bright red color, very uniformly and generally diffused over the whole body, and consisting, on minute examination, of innumerable small points, without any perceptible elevation or roughness,—the color disappearing temporarily on pressure. Its course is rapid, spreading in twenty-four hours over the face, neck, body and extremities, successively, continuously and symmetrically. In small-pox the eruption commences by small papulæ, sensibly elevated above the surrounding skin, usually of the forehead, face and wrists, and is accompanied by a distinct remission of all the febrile symptoms, the pain in the back, headache, etc., and the patient thinks he is getting well. I wish to impress on your minds that the eruption of small-pox may be distinguished by the touch as soon as it appears to the eye, and that this is true neither of measles nor scarlatina. The variolous eruption is a distinctly elevated pustule, prominent and pointed, feeling like a hard nub under the finger, distinct and isolated, and unlike the uniform rash of scarlatina or the patches of measles, neither of which can be detected by the most delicate touch, as elevated, though they may appear so to the eye. I dwell upon this, because every year there are mistakes made by those who should know better; and rubeolous patients are sent to the pest-house, or variolous ones allowed to remain in thickly-crowded tenements.

The measly eruption is easily distinguishable from that of variola and scarlatina in not being raised above the skin—in appearing in irregular patches of two or more papulæ with patches of natural skin between, and by being of a dingy red color. In measles and scarlatina there is no remission of fever until the eruption subsides; while in small-pox there is a remission on the appearance of the eruption, and a recurrence of it at the maturative stage. The measly eruption increases for about forty-eight hours after its commencement on the fourth day of the fever, attains its maximum about the third day (the seventh after the fever sets in), is stationary during the fourth day, and is about two days in disappearing, making the attack, in all, about ten or twelve days in duration—of which four days is occupied by the initiatory fever, and from six to eight days by the eruptive stage, which last usually passes off by a desquamation of the skin, decline of the fever, and abatement of the cough, pain and soreness of the bronchial passages.

With the *treatment* of measles I need not detain you long. You will not, in obedience to the popular error that the greater the disease on the skin, the more thoroughly purified the system; you will not, I say, bundle your patient up in a warm bed in a close, warm room, nor feed him on toddy and hot drinks. You may, it is true, in this way increase the fever and the eruption; but since there is no virus to be eliminated in measles nor scarlatina, as there is in small pox, this is unnecessary, and so far as it tends to make your patient uncomfortable, and exhaust him by too free diaphoresis, it is injurious. You will make your patient's condition comfortable, in a well ventilated room, in a bed with the ordinary amount of clothing on, and not exposed to a current of air. You will regulate his diet, confining it to the plain, simple, easily-digested articles of food and drink: gruels, meat-broths, panadas, arrow-root, toasted bread, and a little solid food, if it is craved; and for drinks, jelly-waters, lemonade, barley-,toast-, or rice-water, if there is no diarrhœa. Little or no medicine is needed, hygienic regulations being usually amply sufficient. The cough and bronchial troubles may be alleviated by the use of the ordinary cough mixture of honey of squills, senega and antimony, blood-root and paregoric, in teaspoonful doses as may be necessary; and to ensure sleep and freedom from restlessness, as well as to lessen the congestion and irritation of the mucous structures, six or eight grains of Dover's Powder and two grains of James' Powder may be given at bedtime. You will occasionally meet cases in which a more active interference will be demanded; cases in which there will be so much soreness behind the sternum and on each side, as to very much distress the patient,—the cough will be attended with an abundant expectoration of a thick, tenacious, ropy mucus, marking a degree of inflammation, supervening on what is ordinarily simple congestion,—the pain in the head will be more violent, the skin dry and husky to the touch,—pulse about 100, and all the febrile symptoms exaggerated. The necessity for medical treatment is now imperative. To your former prescription you may add fʒj of Veratrum Viride to f℥ij of the cough mixture, and give fʒj every four hours, thus lowering the heart's action, and moderating all the symptoms dependent on increased force and rapidity of the circulation. You may also add, for two or three nights, grsij of calomel to the powder of ipecac, opium and antimony, with a view of exciting and increasing the action of the secretory and absorbent systems. There will, probably, be some degree of costiveness, with which it is not well to interfere for two or three days; after which—the use of these medicines being continued meanwhile—a saline cathartic or a dose of oil may be administered, the bowels opened, and the specific alterant and sedative agents omitted, the indications for their use having subsided. In only one of these patients (*No. ten*) has it been necessary to resort to any other than the treatment just given. This patient is of a full, plethoric habit, face flushed and turgid, eyes injected, and conjunctiva and cellular tissue beneath tumefied and congested; the nose also, is swollen and red, as is a space surrounding the alæ, and the pulse is full and bounding in the temporal and carotid arteries. The degree of cephalic congestion here, was so violent as to cause hœmorrhage from the nose, and to such an extent as to require interference. The usual astringents, powdered matico, tannin, etc., were used by the house-surgeon, with temporary success; but we were finally obliged to resort to the Veratrum before the epistaxis was completely checked.

I have detained you, probably, as long as is profitable, on this subject, and will only add a word on a variety of measles, which bears the same relation to the ordinary simple form, as variola does to varioloid, or scarlatina *simplex* to scarlatina *anginosa.* I mean a form of measles occurring in malarious regions, or, in cities, in the children of the poorer inhabitants of the slums and alleys,—children raised in dark cellars, in impure air, in a state of squalid wretchedness and rags and hunger. I am glad to be able to say that I have never met over half a dozen cases of this form in this city. The symptoms differ from those of the type before us, in presenting a livid hue of the face and lips,—pulse generally small and feeble,—extremities cold,—a sense of oppression in the prœcordial region, with laborious breathing, the stomach is irritable, and the intestinal canal shares in the affection, so that the abdomen is tender, and there is both vomiting and purging. The eruption assumes a petechial or ecchymosed appearance, showing a disorganization of the blood, the result both of a malarial poison and of the apoplectic engorgement of the lung, which engorgement is passive, and due to a diminished susceptibility and relaxation of the capillaries of the lung, and not to a direct influx of blood.

Though in civil practice in cities, these cases are rare, and even in malarious districts are not common, yet I should expect to find them occurring frequently in those divisions of our army, posted in the unhealthy swamp lands of the Ohio and Mississippi Vallies, if measles became epidemic among them.

I need not tell you, after recounting such symptoms, that the danger is imminent, and that the promptest measures are demanded. The rapid saturation of the blood with the chlorates or the chlorides, without revolting the stomach, coupled with the exhibition of prompt tonics will be found the most reliable mode of treatment. I have found marked benefit from quinine in three to five grain doses, and chloride of sodium twelve to fifteen grains, repeated every hour during the height of the attack. I have also used the chlorate of potash, instead of common salt, with equally good results.

